# Anaesthetic Management of a Patient With Severe Bipolar Disorder: A Case Report

**DOI:** 10.7759/cureus.111540

**Published:** 2026-06-26

**Authors:** Joshua Nmezi

**Affiliations:** 1 Anaesthesia, Clinical Psychology, Imo State University, Owerri, NGA; 2 Anaesthesia, Pilgrim Hospital, United Lincolnshire Teaching Hospitals NHS Trust, Boston, GBR

**Keywords:** anaesthesia, arrhythmia, bipolar disorder, case report, delirium, lithium, perioperative care

## Abstract

Bipolar disorder is a chronic psychiatric illness that presents unique anaesthetic challenges due to potential drug interactions, mood instability, and perioperative complications. Lithium, a mainstay of treatment, has a narrow therapeutic window and can interact with anaesthetic agents, leading to toxicity, arrhythmias, and prolonged neuromuscular blockade. We report the case of a 45-year-old man weighing 72 kg, with a 10-year history of bipolar I disorder, managed on lithium 400 mg twice daily and olanzapine 10 mg at night, who presented for elective laparoscopic cholecystectomy. Medication adherence was poor, and no preoperative psychiatric review was conducted due to limited access to liaison psychiatry services. Lithium was held for 24 hours preoperatively based on the Royal College of Psychiatrists' guidelines, while olanzapine was continued. Preoperative laboratory findings revealed low lithium levels, deranged urea and electrolytes (U&Es), leucocytosis with neutrophilia, and normal haemoglobin. General anaesthesia was induced with propofol 2.5 mg/kg and rocuronium 0.6 mg/kg and maintained with sevoflurane in oxygen/air. Fentanyl 1 mcg/kg and morphine 0.1 mg/kg were used for analgesia. Intraoperatively, the patient developed haemodynamic instability and supraventricular tachycardia. Emergence was complicated by agitation. Postoperatively, he experienced nausea, vomiting, and delirium, with mood disturbance requiring psychiatric review. He was discharged on postoperative day five. This case highlights the critical importance of preoperative psychiatric assessment, lithium level monitoring, and electrolyte optimisation in bipolar patients presenting for surgery. Multidisciplinary collaboration between anaesthetists, psychiatrists, and surgeons is essential to mitigate risks and optimise outcomes.

## Introduction

Bipolar disorder is a severe mental illness characterised by alternating episodes of mania, hypomania, and depression, affecting approximately 1-2% of the global population [1]. Patients with bipolar disorder often require long-term pharmacotherapy, most commonly lithium, anticonvulsants, or atypical antipsychotics. Lithium remains the gold standard for mood stabilisation but has a narrow therapeutic window and is associated with significant toxicity, particularly affecting the renal, thyroid, and cardiac systems [2]. The toxicities of lithium include nephrogenic diabetes insipidus, hypothyroidism, hypercalcaemia, weight gain, and cardiac conduction abnormalities, including T-wave flattening or inversion and, rarely, sinoatrial or atrioventricular node dysfunction [3].

The surgical patient with bipolar disorder presents multiple anaesthetic challenges. Lithium can potentiate neuromuscular blocking agents, prolong recovery, and increase the risk of arrhythmias [4]. It also affects renal function, leading to electrolyte imbalances that may be exacerbated by perioperative fasting and fluid shifts. Furthermore, the stress of surgery can precipitate mood episodes, and interactions between anaesthetic agents and psychiatric medications are poorly understood [5]. These anaesthetic challenges include haemodynamic instability, arrhythmias, electrolyte disturbances, emergence agitation, and postoperative delirium.

Despite these risks, there is a paucity of literature guiding the perioperative management of bipolar patients, particularly in low-resource settings where access to multidisciplinary care may be limited. Most existing reports focus on lithium toxicity in the surgical setting, but few address the comprehensive perioperative journey [6].

We present the case of a 45-year-old man with poorly controlled bipolar disorder who underwent laparoscopic cholecystectomy and experienced significant intraoperative and postoperative complications. This case highlights critical learning points for anaesthetists and underscores the need for structured preoperative optimisation.

## Case presentation

Patient information

A 45-year-old man weighing 72 kg presented for an elective laparoscopic cholecystectomy for symptomatic gallstones. He was married, lived with his family, and had a 20-pack-year smoking history. He had no previous surgical history.

Psychiatric history

He had a 10-year history of bipolar I disorder, diagnosed by a psychiatrist, with no prior admissions for mania or depression. His maintenance medications comprised lithium carbonate 400 mg twice daily and olanzapine 10 mg at night.

Medication adherence was reported as poor. He had last been reviewed by the psychiatric outpatient clinic four months prior to admission for elective surgery. A referral was made for preoperative psychiatric review, but the patient was not seen due to limited access to liaison psychiatry services. This is a recognised challenge in many healthcare settings, with systematic reviews highlighting the difficulty of accessing multidisciplinary care for patients with serious mental illness undergoing surgery [6].

Preoperative assessment

The patient was assessed by the anaesthetic team on the day before surgery. He demonstrated full capacity to consent and had no acute psychiatric symptoms. Airway assessment revealed Mallampati Class I with good neck mobility [7]. Cardiovascular and respiratory examinations were unremarkable.

A preoperative 12-lead electrocardiogram (ECG) was performed and showed sinus rhythm with non-specific T-wave changes. There were no features of acute ischaemia, prolonged QT interval, or conduction abnormalities. The ECG was deemed acceptable for proceeding with surgery.

His regular medications were managed as follows: lithium was held for 24 hours preoperatively due to concerns about renal function and potential toxicity, while olanzapine was continued. The decision to hold lithium was based on the Royal College of Psychiatrists' guidelines (CR230, 2021), which recommend holding lithium 24 hours before elective surgery and resuming it when renal function and fluid balance are stable [8].

Preoperative laboratory findings

Preoperative laboratory findings revealed a sub-therapeutic lithium level (below 0.6 mmol/L), deranged urea and electrolytes (urea: 12.5 mmol/L, creatinine: 140 µmol/L), and a raised white cell count with neutrophilia (WBC: 14.5 x 10⁹/L, neutrophils: 10.2 x 10⁹/L), though haemoglobin concentration was within normal limits (14.2 g/dL) (Table 1).

**Table 1:** Preoperative laboratory findings

Test	Result	Reference Range
Lithium level	Below 0.6 mmol/L	0.6-1.2 mmol/L
Urea	12.5 mmol/L	2.5-7.5 mmol/L
Creatinine	140 µmol/L	60-110 µmol/L
White blood cells	14.5 x 10⁹/L	4.0-11.0 x 10⁹/L
Neutrophils	10.2 x 10⁹/L	2.0-7.5 x 10⁹/L
Haemoglobin	14.2 g/dL	13.0-17.0 g/dL

The leucocytosis with neutrophilia was investigated. The patient had no fever, no clinical signs of infection, and a normal chest X-ray. A urinary dipstick was negative for infection. The leucocytosis was therefore attributed to lithium-induced benign leucocytosis and physiological stress response rather than infection.

Intraoperative course

General anaesthesia was induced with propofol 180 mg (2.5 mg/kg), rocuronium 50 mg (0.6 mg/kg) for muscle relaxation, and fentanyl 100 mcg (1 mcg/kg). Following induction, the patient was intubated without difficulty, and mechanical ventilation was commenced.

Anaesthesia was maintained with sevoflurane 2-3% in an oxygen/air mixture (FiO₂ 0.5). Morphine 7 mg (0.1 mg/kg) was administered 10 minutes prior to emergence for analgesia.

Approximately 30 minutes into the procedure, the patient developed haemodynamic instability with hypotension (systolic blood pressure: 85 mmHg) requiring vasopressor support. Fluid resuscitation was initiated with 500 mL of intravenous Hartmann's solution prior to vasopressor administration. Despite fluid resuscitation, the hypotension persisted, and a bolus of phenylephrine 100 mcg was administered intravenously, followed by a phenylephrine infusion at 20-40 mcg/min to maintain blood pressure.

The patient also developed supraventricular tachycardia (SVT) with a heart rate of 160-180 beats per minute. This was managed with a bolus of adenosine 6 mg intravenously, which was ineffective, followed by a second dose of adenosine 12 mg, which successfully restored sinus rhythm. The haemodynamic instability and arrhythmias lasted approximately 15 minutes, during which time the patient's blood pressure was maintained with vasopressor support. A 12-lead ECG performed intraoperatively did not show any ischaemic changes.

Intraoperative monitoring included continuous electrocardiography (ECG), pulse oximetry, capnography, and non-invasive blood pressure monitoring. No invasive arterial monitoring was used.

The surgery proceeded and was completed in 90 minutes. At the conclusion of the procedure, neuromuscular blockade was reversed with sugammadex 200 mg (2.8 mg/kg). Emergence from anaesthesia was uneventful initially, but the patient subsequently became agitated in the recovery area and required increased sedation with intravenous midazolam 2 mg.

Postoperative course

Postoperative management included regular intravenous paracetamol 1 g every six hours for pain, along with oral paracetamol 1 g every eight hours once the patient was able to tolerate oral intake. Morphine 2-4 mg was used as required for breakthrough pain. Anti-emetic prophylaxis was administered with intravenous ondansetron 4 mg at induction, followed by a postoperative plan for intravenous ondansetron 4 mg every eight hours as needed for nausea and vomiting.

On postoperative day one, the patient developed delirium, assessed using the Confusion Assessment Method for the Intensive Care Unit (CAM-ICU). He exhibited fluctuating consciousness, disorientation, and inattention. He also experienced mood disturbance with irritability and emotional lability.

Psychiatry was consulted and reviewed him on day two. They assessed his agitation and mood disturbance as an acute-on-chronic presentation related to surgical stress and possible lithium withdrawal (complicated by poor preoperative compliance). Olanzapine was increased to 15 mg at night for short-term mood stabilisation, and lithium was withheld for a further 48 hours until electrolytes and renal function normalised. Daily review by the liaison psychiatry team was arranged and commenced.

Immediate postoperative electrolyte and renal function parameters are presented in Table 2.

**Table 2:** Postoperative day one laboratory findings

Test	Postoperative Day 1	Reference Range
Urea	13.2 mmol/L	2.5-7.5 mmol/L
Creatinine	148 µmol/L	60-110 µmol/L
Sodium	135 mmol/L	135-145 mmol/L
Potassium	3.8 mmol/L	3.5-5.0 mmol/L
Magnesium	0.78 mmol/L	0.7-1.0 mmol/L
Lithium level	0.4 mmol/L	0.6-1.2 mmol/L

Magnesium levels were monitored and remained within normal limits throughout the postoperative period.

Recovery was prolonged. At discharge on postoperative day five, the patient was clinically stable, normotensive, and in sinus rhythm. His delirium had resolved, and his mood was stable. He was discharged with psychiatric clinic follow-up arranged and with advice to resume lithium at his usual dose once renal function had normalised, in accordance with theRoyal College of Psychiatrists' guidelines [8]. He was also advised to attend his general practitioner for repeat lithium level and renal function monitoring within one week of discharge.

## Discussion

This case illustrates the complex perioperative challenges posed by a patient with poorly controlled bipolar disorder undergoing elective surgery. Several critical issues warrant discussion.

Lithium management

Lithium is eliminated almost entirely by the kidneys and has a narrow therapeutic index [2]. Preoperative fasting, fluid shifts, and surgical stress can precipitate lithium toxicity even in previously stable patients [9]. In this case, lithium was held for 24 hours preoperatively, which was appropriate given the deranged renal function and low lithium levels. However, the decision to hold lithium should always be made in consultation with a psychiatrist, as abrupt cessation can precipitate mood episodes [10]. Postoperative monitoring of lithium levels and electrolytes is essential, as was done here. The Royal College of Psychiatrists' guidelines (CR230, 2021) recommend that lithium should be held 24 hours before surgery and resumed when renal function and fluid balance are stable [8].

Electrolyte disturbances and arrhythmias

The patient presented with deranged urea and electrolytes and developed intraoperative supraventricular tachycardia. Lithium can cause electrocardiographic changes, including T-wave flattening or inversion, and rarely, sinoatrial or atrioventricular node dysfunction [4]. Electrolyte abnormalities (particularly potassium and magnesium) may compound these effects. Preoperative correction of electrolytes and baseline ECG should be considered in all bipolar patients on lithium [11]. In this case, the patient's preoperative ECG showed non-specific T-wave changes, which may have been related to lithium therapy.

Haematological findings

The leucocytosis with neutrophilia, in the absence of infection, may represent a stress response or possibly an effect of lithium, which is known to cause benign leucocytosis [7]. Infection was excluded preoperatively, as discussed. This is important, as infection increases anaesthetic risk and would have warranted postponement of surgery.

Emergence agitation and postoperative delirium

Emergence agitation and postoperative delirium are more common in patients with psychiatric illness [12]. Contributing factors may include lithium withdrawal, surgical stress, pain, metabolic disturbances, and interaction with anaesthetic agents. In this case, delirium and mood disturbance required psychiatric input and a prolonged hospital stay. The CAM-ICU was used to assess delirium, providing a validated and standardised approach to diagnosis.

Importance of multidisciplinary care

This patient had no preoperative psychiatric review, despite clear risk factors, due to limited access to liaison psychiatry services. This reflects a common challenge in many healthcare settings. A systematic review of postoperative complications in patients with serious mental illness found that lack of multidisciplinary care is associated with increased morbidity [6]. Guidelines recommend that patients on lithium should have levels checked within the three months prior to surgery, and that a perioperative plan should be agreed between anaesthetist, surgeon, and psychiatrist [8]. This case underscores the consequences of fragmented care.

Comparison with the literature

Similar cases have been reported in the literature. Dominicus et al. described perioperative lithium management in a series of patients, highlighting the risks of arrhythmias and renal dysfunction [5]. Lähteenvuo et al. reported on the real-world effectiveness of pharmacological treatments for bipolar disorder, noting that lithium remains a cornerstone of therapy but requires careful monitoring [13]. In our case, the patient's poor medication adherence and lack of preoperative psychiatric review were key differences from cases where patients were optimally managed. The similarities between our case and the literature include the risks of arrhythmias and the need for multidisciplinary care.

Signs of lithium toxicity

The signs of lithium toxicity include nausea, vomiting, diarrhoea, tremor, ataxia, confusion, and, in severe cases, seizures and coma [2]. Cardiac manifestations include arrhythmias, T-wave abnormalities, and prolonged QT interval. In this case, the patient did not exhibit frank signs of lithium toxicity, but the intraoperative arrhythmias and postoperative delirium were concerning features that warranted close monitoring.

## Conclusions

This case demonstrates that patients with bipolar disorder on lithium undergoing surgery are at significant risk of intraoperative and postoperative complications, including arrhythmias, haemodynamic instability, emergence agitation, and delirium. The adverse events observed in this patient, particularly the arrhythmias and prolonged postoperative delirium, were likely multifactorial, related to lithium withdrawal, electrolyte disturbances, and the physiological stress of surgery.

This case underscores several critical lessons for perioperative clinicians. First, preoperative psychiatric review should be considered essential for all bipolar patients, ideally conducted two to four weeks before elective surgery to optimise medication regimens. Second, lithium levels must be checked within one week of surgery and electrolyte abnormalities corrected preoperatively. Third, a multidisciplinary approach involving anaesthetists, psychiatrists, and surgeons is essential to develop a perioperative plan that minimises risk. Fourth, close postoperative monitoring for delirium, mood disturbance, and signs of lithium toxicity is strongly advised during the postoperative and recovery phase. With appropriate preoperative optimisation and multidisciplinary collaboration, many of the complications seen in this case could potentially be avoided, improving both patient safety and outcomes.
